# How does sutures pattern influence stomach motility after endoscopic sleeve gastroplasty? A computational study

**DOI:** 10.1007/s13304-024-01917-0

**Published:** 2024-07-01

**Authors:** Alice Berardo, Lino Polese, Emanuele Luigi Carniel, Ilaria Toniolo

**Affiliations:** 1 Department of Civil, Environmental and Architectural Engineering, University of Padova, Padua, Italy; 2 Centre for Mechanics of Biological Materials, University of Padova, Padua, Italy; 3 Department of Surgery, Oncology and Gastroenterology, University of Padova, Padua, Italy; 4 Department of Industrial Engineering, University of Padova, Via Venezia 1, 35131 Padua, Italy

**Keywords:** Computational biomechanics, Personalised medicine, Bariatric surgery, In-silico medicine, Endoscopic sleeve gastroplasty

## Abstract

The relatively recent adoption of Endoscopic Sleeve Gastroplasty (ESG) amongst obese patients has gained approval within the surgical community due to its notable benefits, including significant weight loss, safety, feasibility, repeatability, and potential reversibility. However, despite its promising clinical outcomes and reduced invasiveness, there is still a lack of standardised procedures for performing ESG. Multiple suture patterns and stitching methods have been proposed over time, yet rational tools to quantify and compare their effects on gastric tissues are absent. To address this gap, this study proposed a computational approach. The research involved a case study analyzing three distinct suture patterns (C-shaped, U-shaped and Z-shaped) using a patient-specific computational stomach model generated from magnetic resonance imaging. Simulations mimicked food intake by placing wire features in the intragastric cavity to replicate sutures, followed by applying a linearly increasing internal pressure up to 15 mmHg. The outcomes facilitated comparisons between suture configurations based on pressure–volume behaviours and the distribution of maximum stress on biological tissues, revealing the U-shaped as the more effective in terms of volume reduction, even if with reduced elongation strains and increased tissues stresses, whereas the Z-shaped is responsible of the greatest stomach shortness after ESG. In summary, computational biomechanics methods serve as potent tools in clinical and surgical settings, offering insights into aspects that are challenging to explore in vivo, such as tissue elongation and stress. These methods allow for mechanical comparisons between different configurations, although they might not encompass crucial clinical outcomes.

**Supplementary Information:**

The online version contains supplementary material available at 10.1007/s13304-024-01917-0.

## Introduction

Obesity has emerged as one of the most pressing public health challenges of the twenty-first century (13% of the world population is currently involved [1]), affecting individuals, communities, and healthcare systems worldwide. According to data from the World Health Organization (WHO), the prevalence of obesity has nearly tripled since 1975, with an estimated 1.9 billion adults overweight, of whom over 650 million are classified as obese [2]. Defined as an excessive accumulation of body fat that poses a risk to health, obesity not only diminishes quality of life but also contributes to a wide array of chronic diseases and healthcare burdens [3].

Bariatric surgery offers effective and long-lasting solutions for weight loss and improvement of obesity-related comorbidities, by altering the anatomy of the digestive system, reducing stomach size, and/or rerouting the digestive tract to limit food intake and nutrient absorption [3, 4]. Unfortunately, only a small proportion of eligible patients (approximately 1%) currently undergo operations because of the risks and distrust in their management. Endoscopic bariatric therapies have been developed gaining standing because of their minimally invasive nature, reversibility, and applicability in patients otherwise ineligible for bariatric surgery [5–9]. Amongst them, Endoscopic Sleeve Gastroplasty (ESG) has been associated with significant weight loss, excellent safety profile, feasibility, repeatability and reversibility potential. ESG uses a suturing device to create an inner tube or sleeve to reduce the gastric volume without removing tissues by repeatedly stitching the largest curvature of the body.

Initial cases were performed using running stitches, with 6–12 tissue purchase sites, placed in a triangular fashion at the anterior wall, greater curvature, and posterior wall [10]. The suture was pulled to oppose the tissue, and a cinch was placed to secure the plication. Procedure evolved, and to date, several methods of stapling and suturing have been reported without defining a standardised or universal pattern yet. Most of these studies [11–16] proposed a suture pattern proper of the hospital or clinical trial where the procedure was performed. The efficacy (weight loss results and major obesity-associated metabolic diseases evolution) and the safety (major adverse events) of the different patterns and distributions of sutures were studied [12] considering how the number of sutures and total number of stitches applied influenced the results. However, despite the efficacy of these systems, and apparently no differences in the outcomes (weight loss) depending on the suture patterns [11], the influences that these sutures may have on the stomach behaviour has not been clearly identified yet.

In this study, we adopted finite-element modelling to conduct a biomechanical computational analysis aimed at discerning the differences induced by various suture patterns in terms of the mechanical stresses applied to stomach tissues and their corresponding variations in pressure–volume relationships, two key factors that play a role in the mechanotransduction of the gastric receptors and thus satiety. These mechanical quantities cannot be obtained in vivo, contrarily to computational analysis.

The suture patterns analysed consisted in three of the main reported in literature [12]. Rational comparison amongst the different ESG techniques, identifying the critical/more controversial aspects that requires revision (i.e., excessive stress values that could compromise the preservation of an intact tubulisation in the long time) have been reported, revealing another useful aspect of the in-silico medicine for the surgical planning.

## Methods

### Most adopted suture patterns

**Fig. 1:**
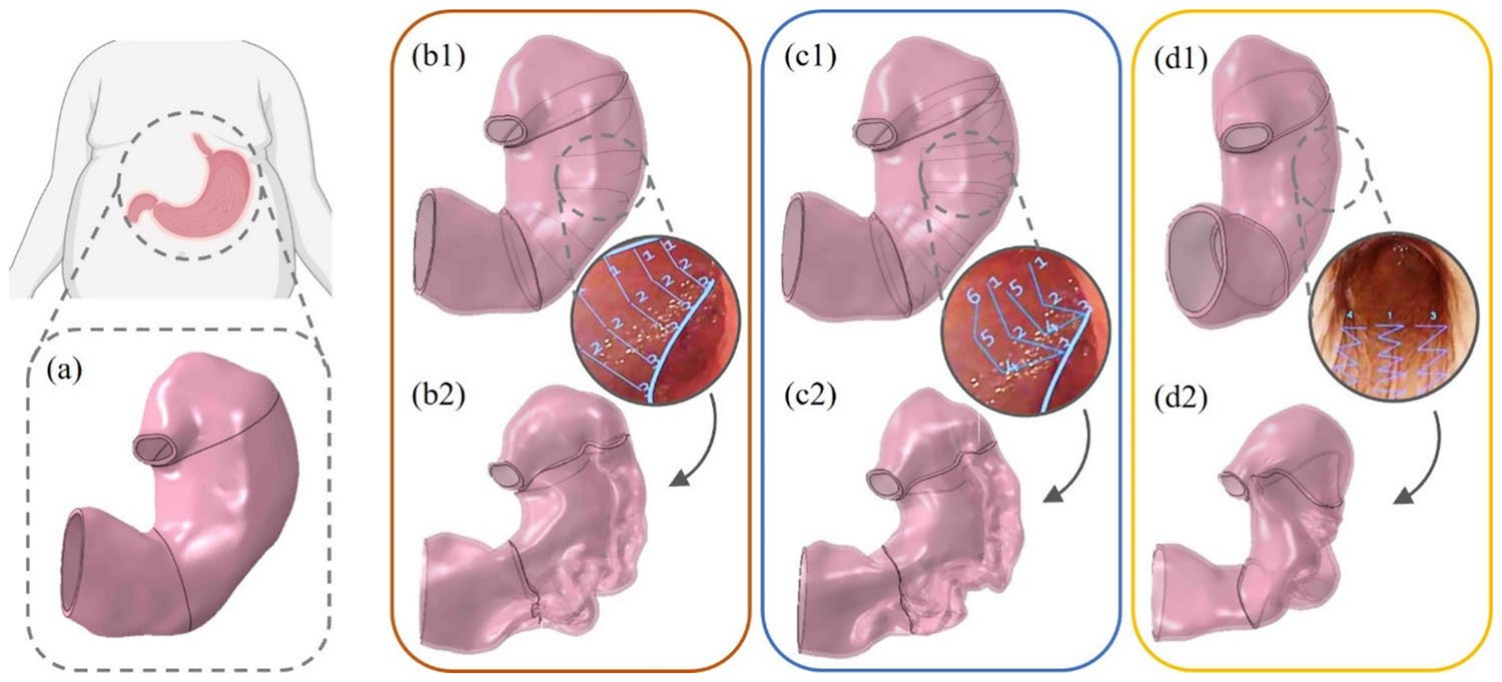
Different types of endoscopic suture patterns used in ESG-Apollo. From a pre-surgical 3D virtual stomach model (**a**), three different suture patterns have been realized, namely (**b**) (TMp, with a “C-shaped” suture path), (**c**) (TBp, “U-shaped” path) and (**d**) (Lp, “Z-shaped” path) (endoscopic images were taken from an open access work [16])

With regard to suture techniques, amongst the variety of possible suture approaches reported in [11], three different patterns have been identified, as reported in [12] (Fig. 1). The first, also called a transverse monolinear pattern (TMp) with a “C-shaped” path (Fig. 1b), is a linear model of suture, starting from the front wall, continuing along the greater curvature and ending on the posterior wall, where the suture is narrow and finalised. The second, a transverse bilinear pattern (TBp) (Fig. 1c), is a triangular suturing pattern performed starting at anterior wall, followed by greater curvature and posterior wall. Then the pattern is repeated, in the opposite direction to 1–2 cm proximal, with the same suture, thus treading a “U-shaped” path. The last one is formed by a longitudinal zig-zag pattern (Lp) with a repeated “Z-shaped” path (Fig. 1d) along gastric body, starting from the antrum and going back up to the fundus. This suture may be repeated three times on the greater curvature, the anterior wall and finally the posterior wall, or can be coupled with the U-shaped for a higher stomach cavity reduction. One single Z-shaped has been analysed in this study.

### Finite element model definition

From an open-source magnetic resonance images file of the abdomen [17], the segmentation of the stomach was performed, followed by the post-processing of the gastric region to generate a double-layer-thickness virtual solid model composed by mucosa layer and muscularis stratum (Geomagics Design X and Solidworks, Dassault Systemes, 2018). Mucosa–submucosa and muscolaris layers were modelled with varying thickness, depending on the stomach region, as was performed in previous studies [18, 19]. The finite-element discretisation (linear hexahedral elements of 1-mm edge size), which serves to describe the response of the gastric wall calculating the mechanical quantities, was performed by means of the finite-element pre-processor Abaqus CAE 2023 (Dassault Systemes Simulia Corp., Providence, RI), resulting in a model with about 730,000 8-nodes hexahedral elements and 857,000 nodes. The full description of the engineering modelling part and the identification of the material parameters assigned to gastric walls were reported in previous works [19–21]. Computational simulations were performed by means of the general purpose code Abaqus Explicit 2023 (Dassault Systemes Simulia Corp., Providence, RI).

### Suture patterns implementation and analysis

The suture patterns that were implemented within this study were three (Fig. 1b–d): (i) the “C-shaped” suture pattern, (ii) the “U-shaped” suture pattern and (iii) a single “Z-shaped” suture pattern.

Wire elements were added to recreate each of the patterns, under the supervision of a bariatric surgeon expert in endosleeves. The linear stiffness of the simulated suture wires was set to 3.5 N/mm after the experimental tensioning of the wires used in surgery (see the Supplementary Materials). The sutures were designed in the gastric region of the corpus, by applying wire features and then, imposing a virtual draught. During the simulation, the first half was dedicated to the closing of the suture (up to 80% of the wire length, thus assuming a 20% of wire remaining within the tissue after wire tensioning), and the second to the simulation of food ingestion by increasing the intragastric pressure from the baseline (assumed as 0 mmHg) to 15 mmHg (approximately 2 kPa). Gastroesophageal and gastroduodenal junctions were fixed.

## Results

The computational results are presented in terms of volumetric capacity (pressure–volume response) and gastric wall tensioning for the three post-ESG stomachs configurations.

After the closing of the sutures, the stomach conformation modified accordingly to the type of suture pattern, thus affecting its volumetric capacity, both at baseline as well as with an increased intragastric pressure. Changes in volume are reported in Table 1.

**Table 1:** Volumetric capacity and percentage of reduction respect to pre-surgical configuration, at baseline and at 15 mmHg of intragastric pressure

	Presurgical stomach	“C-shaped” suture	“U-shaped” suture	“Z-shaped” suture
Baseline	592 ml	158 ml (−73%)	121 ml (−80%)	295 ml (−50%)
15 mmHg intragastric pressure	3412 ml	1460 ml (−57%)	1266 ml (−63%)	2503 ml (−27%)

**Fig. 2:**
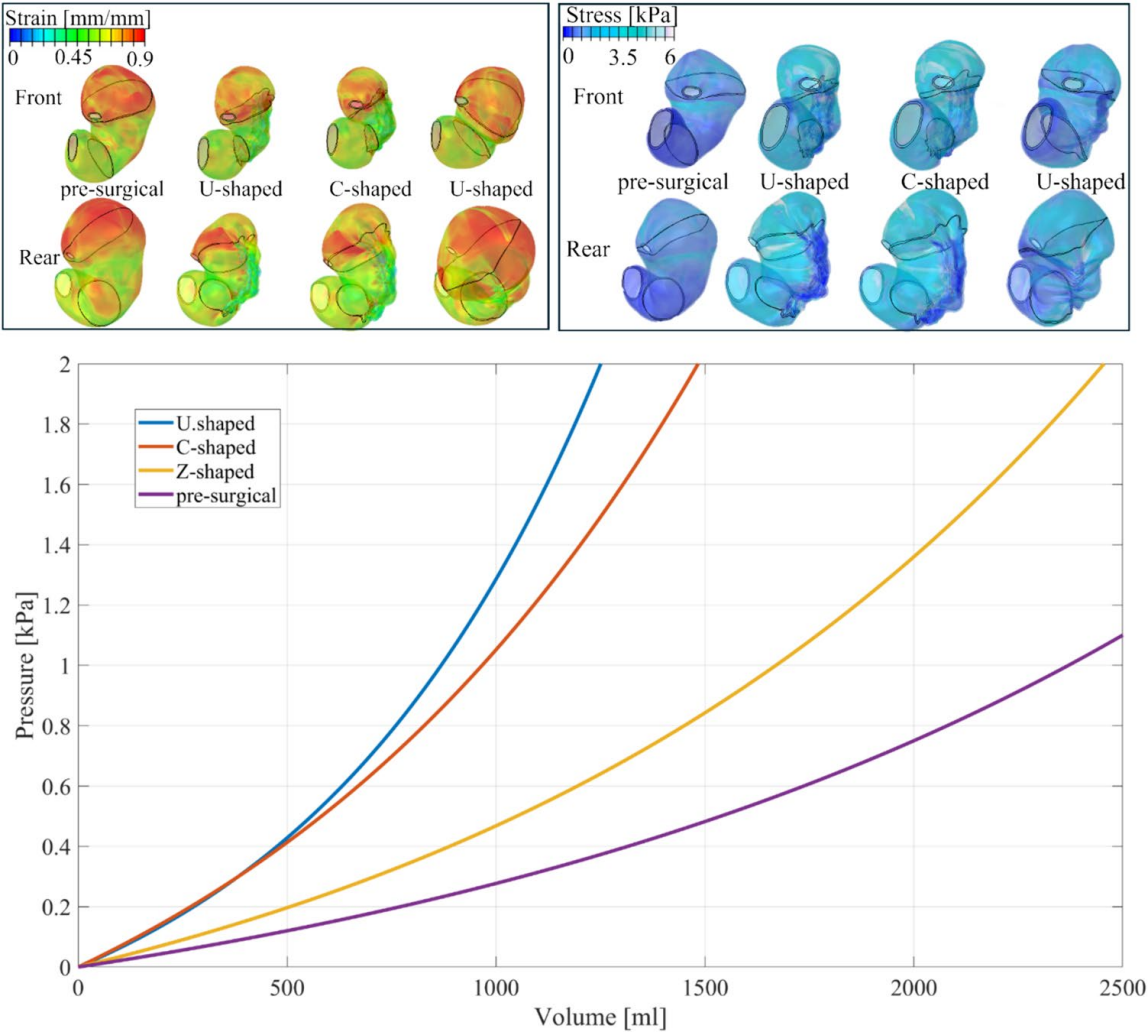
Colormaps of the stomach models in terms of maximum tensile stresses and elongation strains with the application of three types of suture patterns and chart of the pressure–volume behaviours at an intragastric pressure of 15 mmHg. Models are not with the same scale length

The greater reduction operated by the U-shaped sutures influenced the pressure–volume behaviour (Fig. 2), highlighted by the shifting of the curve on the left part of the chart (smaller volumes of food ingestion correspond to higher pressures), however the curve seemed not to significantly differ from the C-shaped one, whilst a halved stomach resulted from a single Z-shaped suture. When a skull-caudal link was placed amongst the circumferential sutures, a shortening of the stomach was seen. The shortening was calculated as the distance of two nodes located on the corpus region, which delimited the corpus from the fundus and the antrum. The shortening is reported in Table 2.

**Table 2:** Shortening respect to pre-surgical configuration, at baseline and at 15 mmHg of intragastric pressure for both the great curvature (l) and the stomach height (h)

		Presurgical	“C-shaped” suture	“U-shaped” suture	“Z-shaped” suture
Baseline	*l* (mm)	173.2	156.3	162.5	137.2
	*h* (mm)	133.2	91.1	83.9	47.2
15 mmHg intragastric pressure	*l* (mm)	267.4	184.0	169.4	146.4
	*h* (mm)	205.0	135.3	120.5	51.4

The strongest shorting was reached with the “Z-shaped” suture pattern, due to the multiple vertical links and to their expansion.

**Fig. 3:**
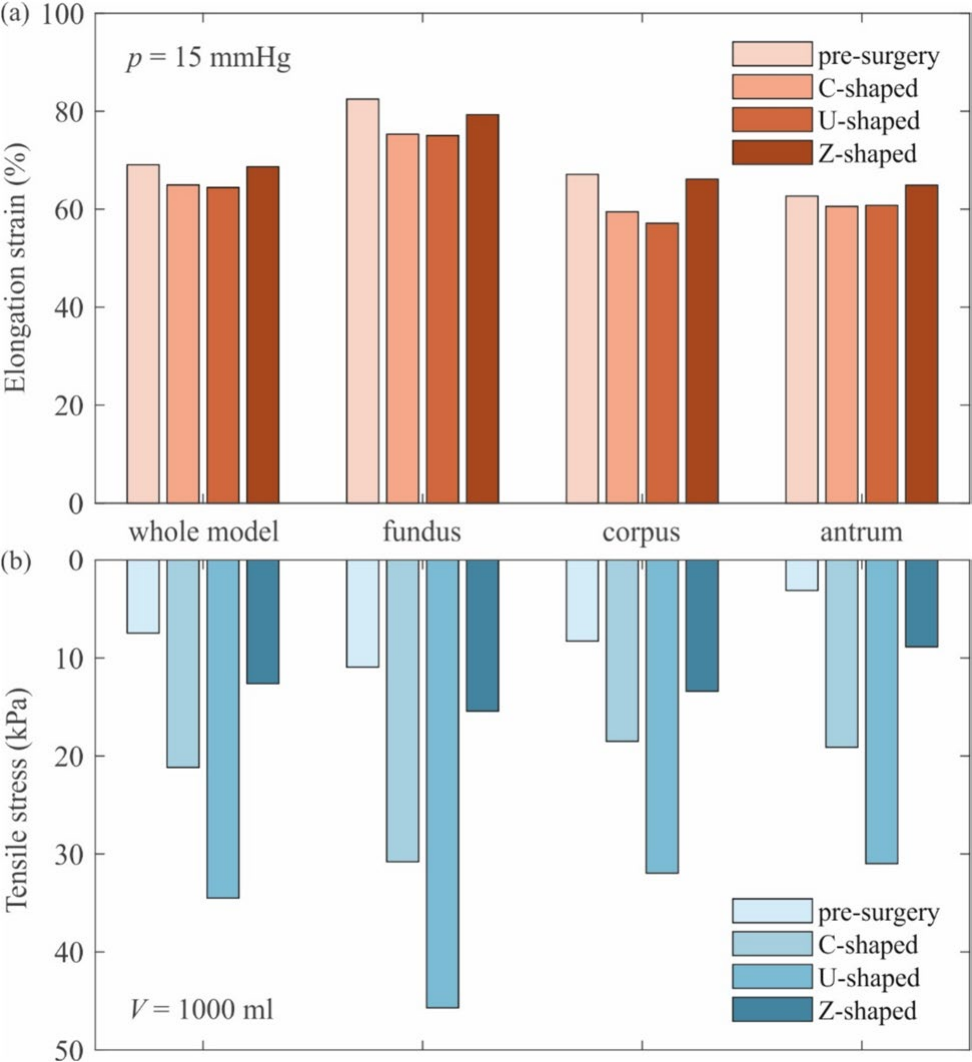
Bars of the elongation strain values obtain at an intragastric pressure of 15 mmHg (**a**) and stress values after a volume increase of 1000 ml (**b**) for the four analysed configurations, differentiated by gastric region

The mean tensile stress values after one litre of ingestion were 21, 35 and 13 kPa for the “C-shaped”, “U-shaped” and “Z-shaped” suture patterns, respectively. The region recording the most stress solicitation was the fundus region, while the lowest stress values were achieved in the corpus for the “C-shaped” and the “U-shaped”, and in the antrum for the “Z-shaped” and the pre-operative (Fig. 3b).

## Discussions

Endoscopic bariatric surgeries are spreading thanks to their minimally invasive procedure and less risk for the patients [22], and the ESG is facing a promising development thanks to the successful clinical outcomes [14, 23, 24]. Several protocols could be adopted to perform the plications, from transverse monolinear (the C-shaped) [14] to greater curvature compression sutures (Z-shaped coupled with U-shaped) [16], since it has been stated that suture pattern does not influence the outcome in terms of weight loss and comorbidity remission at 12-month follow-up [12]. However, nothing is known in the long term, as well as the different mechanical solicitations that a specific suture pattern may induce within the stomach tissues.

For this reason, this study was aimed at mechanically comparing the effects of different suture patterns on gastric wall in terms of stresses and strains and the volumetric reduction of the stomach, thus providing a quantitative description of the implication of ESG procedures, not based only on a posteriori clinical outcome (BMI, TWL, etc.).

When comparing the capacity variation of the stomach volume after the ESG, the U-shaped achieved the greatest reduction (-80% at baseline and -63% at 15 mmHg of intragastric pressure), while the Z-shaped the lowest, (about -50% at baseline and -26% at 15 mmHg of intragastric pressure) (Table 1). These numbers reflexed also within the pressure–volume curves (Fig. 2), where the more the reduction, the more the pressurisation of the stomach at the same reached volume. This could be directly linked to the potential amount of food that can be introduced in the stomach cavity before feeling satiety, thus the U-shaped revealed the strongest food volume reduction, potentially correlated to a faster and greater patient weight loss. However, in the clinical practise the Z-shaped is usually performed combined with other Z-shaped suture patterns [12] or with a U-shaped path [16]. Thus, the resulting post-ESG stomach would result in a greater stomach reduction. However, the aim of this work was to highlight the differences between these suture patterns from a biomechanical point of view, focussing on the variation in stress/strain distributions and stomach shortness after ESG.

The presence of the sutures constrained the tissues in ESG configurations, which was not able to expand itself, decreasing the elongation strain values (Fig. 3a), especially within the corpus region (from an average of 67% of elongation strain to 57% in the presence of a U-shaped suture pattern). However, the antrum region resulted to be the less influenced stomach region, with an oscillation of ± 2% of elongation strain variation with respect to the pre-surgical model.

When considering the tissue tensile stresses, results from the same inflation volume (1000 ml, Fig. 3b) were compared, revealing the U-shaped pattern as the one that solicitates the most the gastric walls (on average more than three times the average pre-surgical stresses). Indeed, this pattern causes a biaxial loading condition, solicitating both the longitudinal as well as the circumferential direction. Moreover, even if the sutures were applied within a unique region (the corpus), all the three regions faced a significant stress increase, with the antrum the most altered zone (stresses up to eight times greater with respect to the pre-surgical stresses at the same stomach volume). As stated before, a single Z-shaped pattern caused a reduced effect also in terms of stresses, even if it should be considered as a partial but already important effect.

**Fig. 4:**
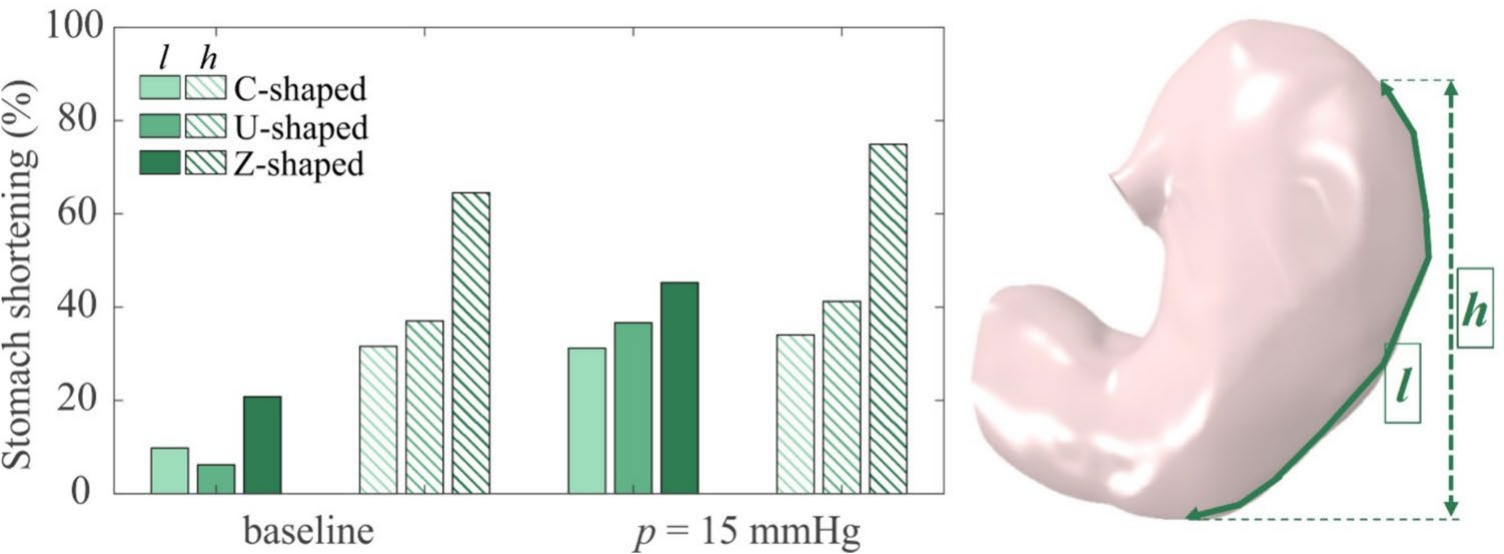
Bars of the stomach shortening measured in two ways, by considering the greater curvature and “vertical” length, for each suture pattern analysed

However, the study of this specific pattern was of interest to quantify the stomach shortness, caused especially by this kind of sutures. Stomach shortness is a result of ESG and leads to a faster stomach emptying and an enhanced stomach stiffness along the longitudinal direction. This latter corresponds to the direction of peristalsis movement (active behaviour) during torniodigestion, thus longitudinal rigidity could affect the stomach primary function due to the interaction with the sutures, that often could lead to tissue damage and suture reopening. In order to monitor stomach shortness, the great curvature length (*l*) or the vertical distance covered by the sutures (e.g. the corpus height, *h*) could be computed and compared (Fig. 4). In this sense, the Z-shaped pattern may cause the strongest stomach reduction, decreasing *l* of about 20%, but especially along the vertical direction, with *h* reduced of 65%. This influence became even more pronounced when the stomach is inflated: being constrained to expand mainly circumferentially rather than longitudinally, when the intragastric pressure reached 15 mmHg the stomach great curvature resulted about one half the pre-operative one, while *h* only slightly modified as reported in Table 2, from 47.2 mm (baseline) to 51.4 mm (74%). This confirmed the Z-suture hinders the physiological gastric volumetric elongation during digestion, and this effect could be even stronger if then the Z-shaped pattern is coupled with other suture patterns.

Results also showed that the other sutures patterns as the C-shaped and the U-shaped could cause stomach shortness (Table 2 and Fig. 4), but up to a maximum of about 40%.

Comparisons with previous finite-element analyses were in agreement with these findings [18, 25, 26], such as an increase in the stomach stiffness (expressed as the ratio pressure/volume) by increasing the number of ESG sutures (the U-shaped is also the pattern with the major number of stiches), reducing the average strain that the stomach exhibits. Also, when including the stomach-specific geometry and a higher intragastric pressure [18], the corpus remains the region with smaller strains compared to the other regions. A first attempt was made to analyse endoscopic procedures in a previous work [20], however the geometry was not patient-specific, and the material parameters were related to porcine gastric tissues.

It should be noticed that this study also acknowledges several limitations inherent in the complexity of the problem. These limitations can be seen in the material parameters used to describe the mechanical behaviour of gastric regions (assumed as an average for the entire stomach regions, more details reported in [18]), the adopted boundary conditions applied to the gastroesophageal and gastroduodenal junctions, and the absence of surrounding organs. All these aspects might collectively contribute to an overestimation of the final volume of the inflated stomach.

Being aware of these limitations, this computational analysis highlighted some major aspects that should be evaluated in the clinical practise, not only to fast reach the strongest stomach reduction (hopefully leading to the reduction of patient weight) but also the mechanical and functional aspects that govern the activation of the mechanoreceptors populating gastric wall. Indeed, as stated by Tack et al. [27], the gastric accommodation reflex (a transient relaxation of the proximal stomach during food intake) has been identified as a major factor which controls meal volume through the activation of tension‐sensitive gastric mechanoreceptors, which similarly mediate gastric filling‐related satiation signals. For these reasons, a mechanical description of the solicitation after food intake could help the clinicians in understating in a deeper way the gut–brain axis, helping bariatric patients in loosening a large amount of weight in an easier and sustainable way, maintaining the improvements in the long terms.

Another information that this model could provide concerns the effect exerted by both gastric filling and peristalsis on the sutures. This could help define which characteristics the suture must have to last longer over time, before failing. The choice of suture type falls on the compromise amongst the following factors: proper gastric reduction, major fundus solicitations and contained distensions at the level of the sutures. From the computational analysis, the authors identified as preliminary best choice a “C-shaped” suture pattern, leaving room for further analyses adding peristalsis in the model.

## Conclusions

The escalating prevalence of obesity represents a public health challenge with far-reaching implications for individuals, communities, and societies worldwide. Bariatric surgery is considered the preferential way to deal with such pathological condition in the most severe cases. ESG is a minimally invasive procedure now widely used for the treatment of patients with moderate obesity. This fairly new procedure is still not completely standardised, particularly regarding the suture pattern. This work highlighted how computational methods can shed light in the major controversies that current empirical methods cannot address, as the level of stress and strain sensed by gastric wall and the quantitative measured of volumetric gastric reduction, with reference to a different chosen suture pattern. In particular, even if no differences have been noticed from a clinical point of view, suture patterns influence the stomach biomechanics, causing variable stress and strain distributions, which potentially could have direct reflexes on the gastric mechanoreceptors. Furthermore, this model can help to better understand the mechanisms underlying suture failure. The in-silico simulations can be further exploited to personalise the surgical procedure, to help the surgeon in intervention planning and to provide a 3D visualisation of the stomach to become familiar with organ steric encumbrance before the endoscopic procedure.

## Supplementary Information

Below is the link to the electronic supplementary material.

Supplementary file1 (DOCX 74 KB)

## Data Availability

The raw/processed data required to reproduce these findings are available upon direct request to the corresponding author.
