# Supplementary material for: How does sutures pattern influence stomach motility after endoscopic sleeve gastroplasty? A computational study
Source: Updates Surg. 2024 Jul 1;76(8):2833–9. doi: 10.1007/s13304-024-01917-0 (PMC11628582; doi:10.1007/s13304-024-01917-0)
Supplement: Supplementary file 1 — Supplementary file1 (DOCX 74 KB) [file 13304_2024_1917_MOESM1_ESM.docx]

**How does sutures pattern influence stomach motility after endoscopic sleeve gastroplasty? A computational study**

**Supplementary Materials**

**Authors:** Alice Berardo^a,b^ , Lino Polese^b,c^, Emanuele Luigi Carniel^b,d^* and Ilaria Toniolo^b,d,^

a Department of Civil, Environmental and Architectural Engineering, University of Padova, Italy

b Centre for Mechanics of Biological Materials, University of Padova, Italy

c Department of Surgery, Oncology and Gastroenterology, University of Padova, Italy

d Department of Industrial Engineering, University of Padova, Italy

* corresponding author: emanueleluigi.carniel@unipd.it

1. **Suture wires stiffness evaluation**

ESG suture wires were tested by means of tensile tests, aiming to evaluate stiffness *k* (N/mm).

ESG sutures were PLL monofilament non absorbable wires 2-0 FILBLOC (Assut Europe, Rome, Italy).

During the experimental protocol, wires were tight longitudinally, with a constant velocity of 0.05 mm/s. The stiffness was computed as the mean slope of the force-displacement curve. Experimentations were performed on two 100 mm length samples.

Figure SM1 reports the linear regions of the force-displacement curves, together with the linear interpolation and the slopes that were obtained from the fit. The experimental results suggested an average stiffness *k* of 3.5 N/mm.

Figure SM1: Force-displacement curves from tensile tests on wires and evaluation of wire stiffness.
